# Living Situation of Juveniles After Secure Residential Treatment: Exploring the Role of Family Centeredness, Child, and Family Factors

**DOI:** 10.1177/0306624X231206517

**Published:** 2023-11-22

**Authors:** Jorinde L. Broekhoven, Lieke van Domburgh, Floor van Santvoort, Jessica J. Asscher, Inge Simons, Annemarieke M. M. M. Blankestein, Gonnie Albrecht, Rachel E. A. van der Rijken, Arne Popma

**Affiliations:** 1 Amsterdam UMC, Department of Child and Adolescent Psychiatry & Psychosocial Care, Amsterdam, The Netherlands; 2 Pluryn, Research and Development Department, Nijmegen, The Netherlands; 3 iHUB Group, Rotterdam, The Netherlands; 4 Child and Adolescent Studies, Utrecht University, Utrecht, The Netherlands; 5 De Banjaard outpatient mental health care service (Youz, Parnassia Group Psychiatric Institute) The Hague, The Netherlands; 6 De Viersprong Netherlands Institute for Personality Disorders, Research, Development & Education, Halsteren, The Netherlands; 7 PI Research, Duivendrecht, The Netherlands; 8 Praktikon, Nijmegen, The Netherlands; 9 Department of Criminology, Leiden University, Leiden, The Netherlands

**Keywords:** juveniles, family centered, systemic intervention, youth care, residential care, closed care

## Abstract

To promote the return of juveniles to a home-like environment (e.g. living with (foster)parents) after secure residential treatment (SRT), it is important to know which factors are related to this outcome. The current study examined which characteristics of the juvenile, family, and SRT, including family centeredness and use of systemic interventions, are related to the living situation after discharge. For 259 juveniles (mean age 15.82 years, 127 girls) in SRT and their parents, questionnaires were administered at admission, discharge, and 6-months follow-up. Furthermore, information about the living situation before and after SRT was gathered. Higher likelihood of living in a home-like setting after SRT correlated with more furlough moments with parents, receiving a systemic intervention, and a shorter duration of the SRT. Systemic interventions during SRT and spending furlough moments with parents may have a positive impact on returning to a home-like situation after SRT for juveniles.

Ideally, juveniles grow up at their home of origin, but if this is not possible, home-like alternatives, such as living with relatives or foster parents, are preferred. However, for a small number of juveniles these home-like settings are not suitable at some point in time, for instance when severe behavior problems interact with complex family problems, causing danger to the juvenile or the environment. When professional help offered at home is not sufficient, residential treatment may be necessary. The most intrusive form of residential treatment in the Netherlands is secure residential treatment (SRT), authorized by a juvenile judge. However, also when placed in SRT, it is important to involve the family in the treatment of the juvenile as they are important attachment figures of the youth, often have parental custody, and as such play an important role in the transfer of treatment effects after SRT (Sunseri, 2004). Placement in secure residential youth care is offered when adolescents show multiple problems which pose a direct threat to the safety of society and/or personal safety, such as self-harming behavior or sexual exploitation (Vermaes & Nijhof, 2014). Although the main reason for placement is the youths unsafety, there is often comorbidity of severe behavioral problems, which indicates the population in residential youth care facilities has many similarities with a forensic youth population. The population in SRT can generally be characterized as having substantial problems, including (externalizing) behavior problems, substance abuse, truancy, and parent-child relationship problems (Dresen et al., 2017). Dirkse et al. (2018) have even shown that in 9% of the referrals, police contacts were the main reason for placement in SRT.

In order to ensure child safety, SRT allows for the use of restrictions when necessary, for instance on contact with others, freedom (e.g., locked doors) and coercion (e.g., fixation) (De Valk, 2019). Due to the many potential restrictions, SRT should only be offered when no other forms of professional care are sufficient, for as short as possible, and aiming for returning to a home-like setting—or at an older age, living independently with support of a social network—and aiming for prevention of repeated re-admission to (secure) residential settings. Thus far, most research on SRT has focused on youth-centered outcomes of residential treatment, such as academic achievement, reduction of behavioral problems, and adaptation in the community (Frensch & Cameron, 2002). The level to which SRT is focused on the family of the youth, family centeredness (FC) and its association with the discharge to a home-like environment as potentially successful contributors to treatment success, has been less studied.

The family centeredness (FC) of the SRT is one of the factors that may be related to successful return to a home-like setting after SRT. Although studies on family involvement in SRT are scarce, family involvement has been associated with positive residential treatment outcomes in different forms of residential care for children and adolescents (Barth, 2005; Geurts et al., 2011; Hair, 2005; Sunseri, 2004), less re-admissions in residential treatment, and to generalization of treatment results after discharge (Casey et al., 2010; Landsman et al., 2001; Merritts, 2016; Robst et al., 2014; Sunseri, 2004). Among detained youth, involvement of parents is reported to have a positive impact on treatment outcomes (Burke et al., 2014; Monahan et al., 2011). As problematic and harmful family interactions are often part of the problems youth are facing when being admitted to SRT (Dresen et al., 2017; Vermaes & Nijhof, 2014), involving parents seems even more important for successful return. By involving parents and important others, and if needed by the use of systemic interventions, the juvenile and the family can learn to stop negative interaction patterns and redress or strengthen their connection. Further, involvement of parents in SRT may promote their confidence in successful reunification after discharge. Therefore, the current paper focuses on the relationship between FC of the SRT and living situation after discharge. As in some cases alternative home like-settings are more suitable than returning to biological parents, the present study also included other home-like settings. The term “parents” in this paper refers to a variety of caregivers: for example biological parents or relatives, adoptive parents, foster parents. In fact, we consider finding other caregivers than biological parents if needed an important part of successfully creating a stable and safe environment for youth to grow up (Landsman et al., 2001). In that case, family centeredness may enhance successful matching with the new caregivers and agreement of the biological parents with the placement, which are important factors related to successful placement in other home-like settings (Haans et al., 2009; Van den Bergh & Weterings, 2010).

Historically, treatment in SRT mainly focused on the youth itself. However, over the past years, the role of the important others around the youth, particularly the family or other care-givers, has gained more attention (e.g., Geurts et al., 2012). This is due to the acknowledgement of the importance of positive family interactions as part of a good pedagogical climate in residential settings (Van der Helm et al., 2018). Parents are likely to be essential agents to generalize treatment effects after discharge and may play an important role in systemic interactions. Family centeredness of SRT can, however, be operationalized in different ways. In this study, three different aspects of family centeredness of SRT institutions were identified: 1) the family centered (FC) attitude and behavior of the group care workers, 2) actual contact between the family and the child such as the frequency of parental visits during SRT and the moments of furlough spent with parents, and 3) the use of systemic interventions (during and after SRT) (Blankestein et al., 2022; Simons et al., 2016).

The FC attitude and behavior of group care workers is an important condition for the effective treatment of juveniles in SRT and family reunification after discharge (Maltais et al., 2019). By thinking and acting from a systemic perspective, for example by involving parents in decision-making and removing barriers for parental visits (Simons, Van Domburgh, et al., 2017), group care workers stimulate juveniles and parents to reconnect and work together. Moreover, group care workers can facilitate and support contact between juveniles and their parents. Although having a family focused attitude seems evident, it is very challenging for professionals in SRT institutions and thus requires explicit focus of the SRT in order to be truly family centered. For instance, parents may not agree with the SRT and, therefore, may not be very cooperative and even hostile in attitude. In addition, youth placed in SRT often show attachment problems to their parents or have suffered trauma in family relationships, making it challenging for group care workers to connect with the family. Also, group care workers in a residential setting have often been trained and have been employed to work with youth. Working with families requires additional skills. This implies that, although it may seem obvious to be family focused, explicit and continuous attention is needed on attitude and believes, but also on practical skills to transfer this into daily practice. Research has previously suggested how FC training and supervision contribute to professionals feeling better capable and voicing less barriers to involve parents (Simons et al., 2017). Thus, FC behavior and attitude are important to measure and cannot be assumed to be present.

Research has shown family-focused programs are effective in promoting parental engagement (Maltais et al., 2019). There are many ways in which this involvement can take place. First, parents should be invited to treatment plan conferences and evaluations. This is standard practice in all SRT. However, how much parents feel part of the treatment plan process can be different as can be the effort that is put into having the parents present. Another form of parental involvement can be parental visits to the youth. Again, this is standard practice, but it may be different how much effort is put into inviting parents and making them feel welcome. Further, parents can be part of the daily living context, for instance by dining or cooking in the residential facility. Finally, youth in SRT have furlough moments to keep a bond and practice for returning home. Research has also shown that parental visits are a strong predictor of reunification (with biological or adoptive parents or a legal guardian) at 6 months after discharge from a residential institution (Lee, 2011). This may be because the contact between juveniles and parents contributes to the motivation of both juveniles as parents to learn from treatment and change destructive patterns.

In addition, given the complex systemic dynamics in the families of these youth that often contribute to the development and escalation of the problem behavior of the child, systemic treatment offered by a trained systemic therapist may be needed for successful SRT, especially when the goal is to return to a home-like setting. In order to be effective, SRT needs to effectively identify which families would benefit from a systemic intervention. In addition, parents or others involved in the family system need to be open to treatment. A FC approach of the residential workers may help to set the stage for effective systemic interventions to be offered. When group care workers think from a systemic perspective, they are more likely to see the importance of—or think of the use of systemic interventions. Systemic interventions aim to reconnect juveniles and parents, provide insight into mutual patterns and how to deal with the destructive ones, in order to decrease the problem behaviors of the juvenile (Carr, 2019).

Besides FC, characteristics of the youth and its system are also likely to influence the likelihood of the youth moving to a home-like setting after SRT and should to be taken into account when studying the relation between FC and living situation after discharge. First, the living situation before SRT possibly predicts the living situation of juveniles after SRT. For example, Den Dunnen (2013) showed juveniles who did not live at home before treatment, were less likely to return home in the six months after treatment. In 2021 in the Netherlands, only 37% of the juveniles lived at home or independently prior to SRT (Jeugdzorg Nederland, 2021). Second, short treatment in a secure setting is associated with less readmissions and more effective treatment results, such as decrease in psychiatric symptoms (Landsman et al., 2001; Leichtman et al., 2001), which may be also indicating a positive effect on returning to a home-like setting after discharge, however, an alternative explanation may be that the problems were less severe in the first place, explaining the quick return home. Third, age may be related to returning to a home-like setting, but results are contradictory. Den Dunnen et al. (2013), for example, found younger children more often were discharged to their home-like situation after residential care, whereas Van Dyk et al. (2014) showed older children and girls are more likely to be discharged to a home-like setting. Fourth, the influence of severity of the behavioral problems of the juveniles has shown contradictory results. For example, Gorske et al. (2003), found presence of more antisocial problem behavior of juveniles decreased the likelihood of successful reunification with the family after placement in SRC. However, Den Dunnen et al. (2012, 2013) did not found a relationship between juvenile problem behavior and discharge to a home-like environment. Fifth, familial problems, such as parental stress may affect the likelihood for juveniles to live in a home-like situation after SRT (Farmer & Wijedasa, 2013). Previous research has shown juveniles from high risk families are more frequently discharged to a restrictive setting (such as Juvenile Justice Institution, residential treatment or drug and alcohol rehabilitation) after SRT (Nijhof et al., 2012). Finally, gender may play a role, although Yeheskel et al. (2020) have shown no overall gender differences in the living situation after SRT placement. Nevertheless, gender may have an indirect relationship as girls in SRT show more severe problem behaviors and are more likely to come from dysfunctional families than boys (Nijhof et al., 2012) which in turn may influence the likelihood of being discharged to a home-like setting.

The aim of the current study is to focus on the association between-the three components of FC (behavior and believes, parental involvement, and systemic interventions) and living in a home-like environment six months after discharge from SRT. A population of juveniles who were placed in one of the seven SRT centers who participated in this study, was included. Based on what is described above, we expected family centeredness, in terms of FC attitude and behavior of group care workers, parental visits, furlough moments spent with parents and the use of systemic interventions, will increase the likelihood for juveniles to live in a home-like environment after SRT. Further, we aimed to study the correlation between other potential factors such as living situation prior to placement, duration of treatment, youth characteristics and parental stress, and living situation 6 months after discharge. Finally, we analyzed which factors are most important when combining all these potential correlates.

## Method

### Participants and Procedure

**Figure 1.**
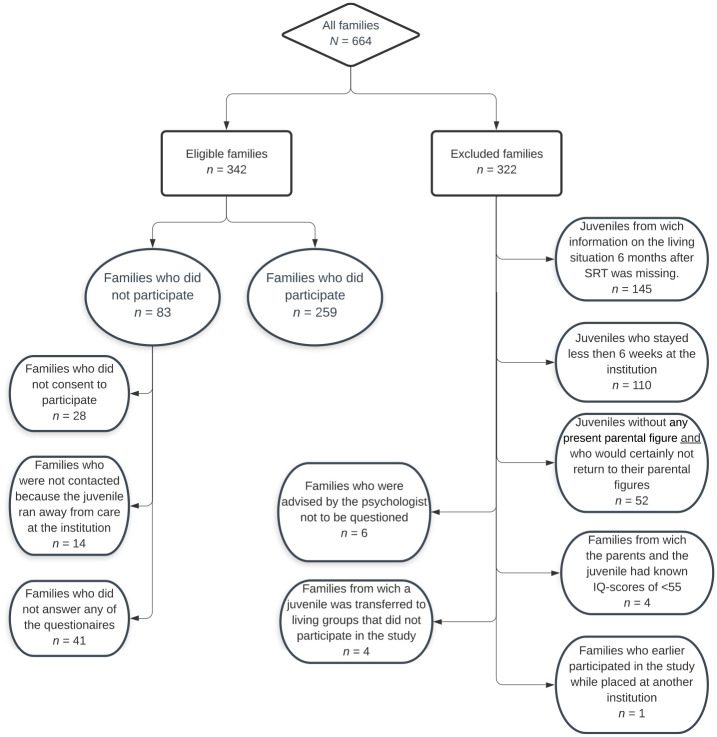
Flowchart of participants.

In this prospective study, 664 juveniles were placed in one of the seven participating SRT institutions between February 2016 and June 2018, and as such were eligible for participation. However, some families were excluded from this study, because data on follow-up were missing, because the duration of their stay in SRT was less than 6 weeks, or because no parent figures were present. Figure 1 (see next page) gives a detailed description of the reasons for exclusion. The analyses of this paper are based on a total of *N* = 259 families.

Of these 259 juveniles, 49% were female. The majority had a Dutch background (59.5%) and for 40.5% of the juveniles, at least one biological parent had a migrant background. The average age was 15.82 years (*SD* = 1.22; range 12–18 years).

Parents filled out questionnaires at baseline when entering the SRT (T1) and at the end of the placement (T2) about the problem behaviors of their child and about parenting stress. At follow-up (T3), 6 months after the end of the placement, parents or juveniles were asked about the living situation(s) of the juvenile in the past 6 months. Group care workers answered questionnaires at the end of the placement regarding parental visits and furlough moments of the juveniles during placement. Furthermore, every 6 months, group care workers filled out a questionnaire about the level of the family centeredness of their living group.

Depending on the rules and procedures of the institution, all families actively (through a consent form) or passively (through an information form which stated they could inform the mentor or the coordinating therapist by email or verbally if they objected to participation) consented to participate. The study was reviewed by the Medical Ethics Review Committee of the VU University Medical Centre. They concluded that the study falls outside the realm of the WMO (Dutch Medical Research in Human Subjects Act) and that it conforms to Dutch law, including ethical standards.

### Setting

In The Netherlands, annually about 1,500 juveniles are being placed in Secure Residential Treatment (JeugdzorgPlus Plaatsingsgegevens, 2021a). As of 2005, these facilities are available to facilitate compulsory treatment for juveniles as a child protection measure for juveniles with severe (externalizing) behavior problems who were at severe risk, and in need of protection against themselves or against others. Secure residential youth care is one of the most restrictive forms of residential youth care in the Netherlands (Harder, 2011). Aside from offering treatment, attention is paid to the pedagogical climate and schooling of the youth. A good pedagogical climate offers support and responsivity, opportunity to grow and develop, structure and rules, positive interactions between adolescents, safety, and encourages interactions between adolescents and their parents (Van der Helm et al., 2018). Increased attention has been paid to family centeredness in SRT over the last years in different ways in different facilities, ranging from training staff in family-centered focus during stay to adding systemic therapists to the staff. Seven of the—at that time—13 facilities for secured residential treatment in The Netherlands participated in the current study.

### Instruments

#### Family Centeredness

##### Family Centered Attitude and Behavior

A questionnaire that has been used in previous research on family centered care in juvenile justice institutions (Simons et al., 2016) was adapted into a 31-item questionnaire to measures family centered attitude and behavior of group care workers. Adaptations were minor: three questions on cultural background were added and some terms specific for juvenile justice institutions were changed to meet SRT setting. More details are available upon request by the first author. The questionnaire consists of 14 questions that can be answered on a 5-point scale (1 = “Never” to 5 = “Always”; later recoded to a 10-point scale), for example, “For parents of each juvenile a tour of the institution is provided,” and 17 questions that can be answered on a 10-point scale (1 = “Completely disagree” to 10 = “Completely agree”), for example, “By working together with the parents, I understand the problem behavior of the juvenile better.” The reliability of the total scale was acceptable (range of Alphas across the measurement moments: α = .62–.86).

The questionnaire was completed by the group care workers of each SRT group every 6 months, with a maximum of six measure moments. All adolescents in one residential group were attributed the same FC score. Further, as scores of FC differed little over time per team, we calculated the mean of all measurement moments of the residential group. A higher score reflects a higher level of family centered attitude and behavior.

##### Parental Visits and Furlough Moments

Parental visits and furlough moments spent with parents were based on an interview with the mentor of each juvenile. In SRT each juvenile is assigned a group care worker as their personal mentor. The mentor is the linking pin in the communication between the youth, the group care workers, other involved professionals, and the parents concerning day to day practice. Parental visits were scored based on the following questions: “Did parents visit the juvenile at the institution?” (if yes, how many times a week), “Did a family intake take place?” (yes or no), “Did parents attend treatment plan discussions?” (yes or no), and “Did parents visit the institution to participate in treatment interventions for the adolescent?” (yes or no) “Did parents visit the institution for any other activities (e.g., a day or evening that was organized for parents, cooking activities, or joining for dinner)?” (yes or no). The answers on the first question have been dichotomized into “low involvement” (i.e., less than once a week) and “high involvement” (i.e., once a week or more). Finally, a mean score of all the dichotomous answers was calculated, with a higher score indicating more parental visits (range 0–1). Furlough moments spent with parents was measured by the question: “Did the juvenile spent furlough moments with parents?” (yes or no).

##### Systemic Interventions

Of the eligible families, 70% met the inclusion criteria for a systemic intervention as commonly used protocolized systemic interventions, indicating the family could potentially benefit from a systemic intervention in addition to SRT. The current study used a broad definition of systemic interventions, covering various interventions consisting of family therapy (Carr, 2019). Information about the use of systemic interventions during and after SRT was gathered on T2 by asking the mentor of the juvenile and on T3 by asking the parents and/or the juveniles if a systemic intervention was used. Systemic interventions were scored as such when an additional intervention was offered to the family on indication as addition to SRT. All kind of systemic interventions were included in this study, for example Multidimensional Family Therapy (MDFT; Liddle et al., 1991), Multisystemic Therapy (MST; Henggeler et al., 2009) and Systemic Therapy (ST; Savenije et al., 2010) (see Blankestein et al., 2022, for a more detailed overview). The variable was dichotomized as *systemic intervention yes (1) or no* (0).

#### Problem Behavior

The problem behavior of the juveniles was assessed using the Brief Problem Monitor for Parents (BPM-P; Achenbach et al., 2011). The questionnaire consist of 19 items, for example “Disobedient at home”. Answers can be given on a 3-point scale ranging from *never* (0) to *often* (2). A total score was calculated with a higher score indicating more problem behavior from the perspective of the parents. The reliability of the total scale was very good (T1 α = .82 and T2 α = .88). The parent-reported problem behavior concerns behavior observed by the parents at home (T1) and or during visits and on furlough moments (T1 if juvenile was not living at home prior to placement in SRT and T2).

#### Parenting Stress

The degree of parenting stress experienced by parents was measured with the Parenting Stress Questionnaire [Opvoedingsbelasting Vragenlijst] (OBVL; Vermulst et al., 2015). The questionnaire consists of 34 items. Answers ranged from *not at all true* (1) to *completely true* (4). The total parenting stress scale was used in the current study, with a higher score indicating a higher level of self-reported parenting stress. The reliability was very good in this study (T1 α = .91 and T2 α = .92).

#### Demographic Characteristics

Demographic characteristics of the juveniles were administered during the SRT from case files and included age at admission, gender, cultural background, living situation before SRT and the duration of SRT in months. Cultural background was coded into “Dutch” if both biological parents were born in the Netherlands and “migrant” if at least one of both biological parents was born elsewhere. The living situation before SRT included in the analyses was recoded into “living in a home-like environment” (e.g., with (adoptive) parents or relatives, foster parents or in a “gezinshuis” or family-home) and “living in a non-homelike setting” (e.g., open residential setting, crisis care, another secure residential setting, Juvenile Justice Institution, and other places such as hospital or rehabilitation clinic). A “gezinshuis” or family-home is a small scale facility where a couple (of whom at least one is a paid professional) provides care for youth in a family environment.

#### Living Situation During 6 Months After SRT

At the end of the placement and six months after placement, parents or juveniles themselves were asked about the living situation of the juvenile in the period six months after SRT. Juveniles who lived both in a home-like environment and in a residential setting (*n* = 11) were excluded from the analyses, because we did not know in which order the living situations followed each other. However, juveniles who moved between living situations, but within the same category (for example a juvenile who first lived in a foster home after SRT and then moved to his biological parents, was coded as living in a home-like environment) were included in the analyses.

### Statistical Analysis

#### Missing Data

Due to non-response of families, the data contained missing values. In order to asses patterns of missing data, families with and without missing data were compared through *t* test analyses (for continuous variables) and Chi square analyses (for dichotomous variables). In total, 61.4% of the families had missing data on at least one variable. The variable problem behavior had 27% missing data on T1 and 31% on T2, the variable parental stress had 30% missing data both on T1 and T2, parental visits had 8% missing data and furlough moments 10%. Analyses showed that in families with missing data, more juveniles had an migrant background (χ^2^[1] = 5.44, *p* = .020) when compared with families without missing data. However, analyses showed that on the other baseline variables families with and without missing data did not differ.

To enable the use of data of all 259 families, missing data were imputed 40 times in IBM SPSS Statistics version 26 using Multiple Imputation (Regression method).

#### Correlation and Regression Analysis

First, we used Spearman correlation analysis to examine the bivariate relationship between the outcome variable (i.e., living situation in the six months after SRT) and the predictor variables (i.e., age, gender and cultural background of the juvenile, duration of SRT, FC attitude and behavior, parental visits, furlough, receiving a systemic intervention, living situation before SRT, problem behavior (T1 and T2), and parental stress (T1 and T2)).

Next, predictors with a significant association to the outcome variable were combined in a logistic multiple regression analysis to analyze the robustness of the relationship between the predictor variables and the outcome. All predictor variables were simultaneously included in the regression analysis.

All analyses were two-sided with a significance level of *p* < .05.

## Results

### Descriptive Analyses and Group Comparison

Descriptive statistics of baseline and predictor variables are shown in Table 1. Of the juveniles, 40.2% lived in a home-like environment before SRT, of which 39.0% with biological parents or relatives and 1.2% in a family-care home or foster home. Of the remaining 59.8% who did not live in a home-like setting before SRT, 56.7% lived in a residential setting (e.g., open residential setting, crisis care, secure residential setting, rehabilitation clinic), and 3.1% in a Juvenile Justice Institution.

**Table 1.** Descriptive Statistics.

Variables	Baseline (T1)	End of SRT (T2)
	%	%
Living in a home-like environment		
Yes	40.2	44.4
No	59.8	55.6
Furlough moments with parents		
Less than once a week		36.1
Once a week or more		63.9
Systemic intervention		
Yes		31.7
No		68.3
	*M* (*SD*)	*M* (*SD*)
Family centeredness		7.21 (0.21)
Parental visits		0.43 (0.20)
Juvenile problem behavior	19.59 (6.62)	13.97 (7.54)**
Parental stress	70.48 (15.29)	63.30 (15.38)**
Duration of SRT (in weeks)		29.75 (18.80)

** Significant difference between T1 and T2 at *p* < .001.

In the six months after SRT, 44.4% of the juveniles lived in a home-like environment or lived independently. Of these 44.4%, 37.1% lived at home, 4.2% independently, and 3.1% in a foster home. Of the remaining 55.6% of juveniles who did not live in a home like setting in the 6 months after SRT, 50.2% lived in a residential setting (i.e., open, secure, sheltered housing), 3.1% resided in a Juvenile Justice Institution, and 2.3% were homeless.

Since the group of juveniles who lived independently (*n* = 11) was small, we decided to include these juveniles within the group of juveniles who lived in a home-like environment after SRT, because these juveniles live in a *‘normal’* situation and not within residential care. The characteristics of these 11 juveniles were very similar to those in the full sample except for a lower amount of furlough moments with parents during SRT than juveniles in the whole sample (χ^2^[1] = 5.22, *p* = .022).

The majority of youth (63.9%) spend furlough moments at home at least once a week, while about one-third (31.7%) of the families received an additional systemic intervention. Regarding FC the *SD* was rather low (*SD* = 0.21). Most variation was found on item level on the item “parents are difficult to work with” (*SD* measure moment 1 = 2.00), and the subscale “hindering thoughts” (*SD* on first measurement 1 = 1.33).

#### Results of Spearman Correlation Analysis

In order to decide which variables to include in the regression analysis, we ran correlations. As Table 2 shows, characteristics of the SRT such as family centered attitude and behavior, parental visits, furlough, duration of the SRT, and systemic interventions correlated with youth living in home-like settings after SRT. In contrast, individual characteristics as age, gender, and cultural background did not correlate with living situation after SRT. Higher levels of problem behavior of the child and parental stress were associated to lower likelihood of living in a home-like setting after SRT only when measured at the end (T2) of SRT.

**Table 2.** Spearman Correlations.

Variables	1	2	3	4	5	6	7	8	9	10	11	12	13	14
1. Living situation after SRT														
2. FC attitude and behavior	.257**	—												
3. Systemic intervention	.361**	.315**	—											
4. Parental visits	.154**	.154*	.244**	—										
5. Furlough moments	.293**	.238**	.177**	.240**	—									
6. Cultural background	−.047	−.007	−.186**	−.040	−.014	—								
7. Age	−.006	−.081	−.156*	−.049	.042	−0.50	—							
8. Gender	.009	.022	0.80	.122	.024	.073	−.050	—						
9. Duration of SRT	−.342**	−.194**	−.237**	.063	−.197**	.143*	−.147*	.019	—					
10. Living situation before SRT	.203**	.103	.272**	.172**	.133	−.081	−.018	.000	−.225**	—				
11. T1 problem behavior	−.126	−.111	.025	−.071	−.070	−.121	−.049	−.065	.115	.046	—			
12. T2 problem behavior	−.180**	−.046	−.086	−.162*	−.109	−.081	.077	−.118	0.18	.050	.469**	—		
13. T1 parental stress	−.109	−.095	.067	−.039	−.079	−.163*	−.005	.053	.069	.004	.479**	.208**	—	
14. T2 parental stress	−.192**	−.094	−.017	−.019	−.070	−.061	.062	.041	.020	−0.22	.240**	.484**	.402**	—

*Note.* 1. Living in a home-like environment after secure residential treatment (0 = no, 1 = yes); 2. Family centered attitude and behavior of group care workers; 3. Use of systemic intervention (0 = no, 1 = yes); 4. Parental visits; 5. Furlough moments spent with parents (0 = no, 1 = yes); 6. Cultural background of adolescent (0 = Dutch background, 1 = migrant background); 7. Age; 8. Gender (0 = male, 1 = female); 9. Duration of secure residential treatment; 10. Living in a home-like environment before secure residential treatment (0 = no, 1 = yes); 11. Juvenile problem behavior at T1; 12. Juvenile problem behavior at T2; 13. Parental stress at T1; 14. Parental stress at T2.

* p < .05. **p < .01.

Moreover, Table 2 shows that the family-centered attitude and behavior of SRT group care workers significantly correlated with more use of systemic interventions, more parental visits, and more furlough moments spent with parents.

#### Results of the Multivariate Logistic Regression Analysis

When combining the statistically significant predictor variables in a logistic regression analysis, only the use of systemic interventions, furlough moments spent with parents, and the duration of SRT significantly predicted the living situation in the 6 months after SRT (see Table 3). Specifically, the use of systemic interventions, more furlough moments spent with parents, and a shorter duration of SRT increased the likelihood for a juvenile of living in a home-like environment after SRT.

**Table 3.** Coefficients of the Model Predicting the Living Situation in the 6 Months After SRT.

Multivariate logistic regression analysis					
	*B*	Lower	OR	Upper	*p*
Constant	−4.292		0.014		.435
FC attitude and behavior	0.810	0.507	2.247	9.961	.286
Systemic intervention	**1.214**	**1.697**	**3.369**	**6.689**	.001**
Parental visits	0.789	0.373	2.201	12.995	.383
Furlough moments with parents	**.883**	**1.202**	**2.418**	**4.862**	.013*
Duration of SRT	−**0.033**	**0.949**	**0.967**	**0.986**	.001**
Living situation before SRT	−0.305	0.392	0.737	1.387	.344
T2 juvenile problem behavior	−0.026	0.922	0.974	1.029	.343
T2 parental stress	−0.020	0.955	0.980	1.005	.122

*Note.* Significant results are presented in bold.

* *p* < .05. **p < .01.

## Discussion

The current study examined which characteristics of the juvenile, family, and SRT itself are associated with living situation after discharge from a SRT at 6-months follow-up, given the importance of growing up in a home-like setting after SRT. Overall, almost half (44.4%) of the youth lived in a home-like setting 6 months after residential treatment. In line with our hypotheses, characteristics of the SRT related to family centeredness correlated with youth living in home-like settings after SRT. In contrast, individual characteristics such as age, gender, and cultural background did not predict the living situation after SRT. Higher levels of problem behavior of the child and parental stress were negatively associated with the likelihood of living in a home-like setting after SRT only when measured at the end (T2) of SRT while living in a home-like setting prior to SRT positively associated with the likelihood of living in a home-like setting after SRT. When studied together, only furlough moments spent with parents, receiving a systemic intervention during SRT, and a shorter duration of the SRT independently contributed to the likelihood of living in a home-like setting at least six months after SRT.

Although family centered attitude and parental visits correlated positively with living in a home-like setting after SRT, only furlough moments spent with parents and receiving a systemic intervention independently contributed to a higher likelihood of living in a home-like setting after SRT. This could be explained by several mechanisms. First, having a family centered attitude may in itself not directly contribute to living in a home-like setting but may rather set the stage for other interventions that do. For instance, a family centered attitude is likely to promote involvement of parents and thereby furlough moments with parents. In line with this, the items “finding it difficult to work with parents” and “having hindering thoughts” showed most variation among teams. In addition, a family centered attitude is likely to improve the bond with parents and thereby may promote parental motivation to engage in systemic interventions. Finally, a family centered attitude of the involved professionals may also increase the likelihood the professional will identify a family as being eligible for systemic intervention and increase motivation of families themselves. In line with these explanations, a family centered attitude is associated to all these characteristics. Parental visits were not independently correlated with living in a home-like setting after SRT. These visits to the facility were rather common, and may be of limited preparational value for returning home, whereas having furlough moments is more important as preparation for returning to a home-like setting. So, although family centered attitude may be important to pave the way, it is systemic treatment and practice during furlough that is essential for returning to a home-like setting after SRT.

This study found little evidence relating individual characteristics (i.e., age, gender, and cultural background) and level of youth problems and family stress at admission to living situation after SRT. This may be explained by a selection effect that takes place earlier: possibly, those families participating in systemic treatment are also the families where the likelihood to return home is higher. Given the many, often static, risk factors youth in SRT have been exposed to, it may be a realistic outcome that returning to a home-like setting is not possible for all involved. Although not independently correlated when all correlation variables were taken into account, problem behavior at T2 and parental stress at T2 did correlate with a lower likelihood of living in a home-like environment after SRT when studied separately. One could expect these factors would be related to duration of SRT or furlough moments with a lower level of behavioral problems and parental stress correlating to shorter duration and more furlough moments. However, such correlations were not found. Duration of SRT and furlough moments were correlated with FC and receiving a systemic intervention. This is not surprising as these interventions aim for adolescents to return home as soon as possible or to live independently after placement (Rovers et al., 2019; Trupin et al., 2011). However, the current study did not specifically look into mechanisms behind decisions on furlough moments and SRT treatment duration. In addition, a rather large part of the eligible youth and families were excluded from the study because data on their living environment six months after SRT were missing. Possibly, less well functioning youth and/or parents or youth and parents who had a more negative experience and thus attitude to SRT may have dropped out as they were the most difficult to get information on 6 months later. This may have caused a selection effect. Finally, duration of SRT itself could have had a negative impact on the likelihood of living in a home-like environment due to hospitalization or other detrimental effects of SRT. Future research into reasons and mechanisms influencing duration and furlough is of high importance as it may help SRT to improve and diminish negative effects as much as possible. In addition, future research would benefit from not focusing on SRT on its own but rather as SRT as part of a chain of interventions or trajectory.

### Limitations

There are some limitations, which may have affected our results. First, although we included living situation prior to SRT as a variable, we did not extensively look into care history prior to SRT. Youth placed in SRT often have a long history of placements in different residential and home-like settings (Vermaes & Nijhof, 2014) which may have influenced FC and the likelihood of returning to a home-like setting after discharge. Second, parent-reports were used to measure parental stress and behavior problems, respectively. As we used parent reports, scores on behavior problems were based on behavior as observed by parents during furlough moments or visits. This may have been different from the behavior and change over time in behavior as observed by professionals of the SRT. This may have affected the association with returning to a home-like setting. In the same regard, self-report measures on FC were used instead of actual observations. Future research could benefit from incorporating more objective measures of behaviors and attitudes of both youth and professionals. Third, in our study we did not differentiate in type of pathology underlying potential behavior problems of juveniles. It may be that different types of pathology such as trauma, attachment, autism, and different combinations of these pathologies result in a different likelihood of living in a home-like setting. Furthermore, we did not focus on specific internalizing forms of problem behavior, such as anxiety, depression, auto-mutilation, and suicidality in this study. These behaviors, may have significant impact on the safety of the child and its environment and require intensive care and attention, which may make it less likely to be able to live in a home-like setting after SRT treatment. Fourth, although we focused on living in a home-like environment after SRT, we did not look at the quality of life or more specifically into the wellbeing of the youths and/or their families and the quality of their relationship. It could, for instance, be that some youth are living with their family but have a poor relationship while others live in a residential home and have a supportive relationship.

Finally, we did not focus on the trajectory after leaving the SRT, whether it succeeded as planned and how it is related to family centeredness. Family centeredness may for instance improve the assessment of who can benefit from what type of trajectory after release from SRT (home, open residential facility, etc.), and from a systemic intervention and the agreement between professionals, youth and parents on these trajectories. Future research may focus on the influence of family centeredness on successful trajectories and on the level of shared decision making.

## Conclusion and Implications

Despite the limitations, the present study clearly showed the importance of not forgetting the families while the youth is involved in SRT. Especially the use of systemic interventions is associated with the likelihood of returning to a home-like setting. In the current study, about 30% of the families received a systemic intervention while about 70% met inclusion criteria for such an intervention Considering the positive impact of systemic interventions on living in a home-like setting after SRT, it might be worth focusing more on identification of those families who could benefit from systemic interventions. These families should be offered such interventions and be motivated to participate in these interventions. Earlier studies have shown high levels of family problems in families of juveniles in (secure) residential care (Van Dam et al., 2010). Several mechanisms may hamper optimal systemic care. First, the SRT facility but also the Youth protection officer who mandates the treatment may have a lack of focus on the system as a whole. By working together in keeping the focus on the system of the youth, families may become engaged in earlier stages and possibly more families may become motivated to work towards recovery with the youth and the SRT facility. Second, it proved to be difficult to offer the treatment in SRT because of several reasons such as lack of therapists, long travel times between the institution and the family, and lack of funding as some municipalities do not fund systemic treatment when residential care is offered. Practical issues such as these should be identified, listed, and placed high on the political agenda and on the agenda of the facilities and municipalities. Finally, although this cannot be concluded based on this study, given the large amount of risk factors present, and the often long history of youth care of the families involved, there may be challenging getting a good alliance with families which is needed to accept systemic interventions. This may be particularly the case in families with a migrant background as a migrant background was negatively correlated with receiving a systemic intervention. Although we should be very careful to not overinterpret these correlations given the number of correlations run and given it was not our main aim of study, it could be relevant to look into potential undertreatment of migrant families in future research.

The present study showed which factors predict returning to a home-like setting after residential treatment. Despite our expectations, actually, not personal characteristics were most predictive, but how furlough was spent and whether systematic interventions were used. In order to give all youth involved equal chances, it may thus be important to determine which factors predict which families are receiving systemic interventions and what determines which youth gets to spent furlough moment with the parents.
